# Efficacy of Low-Molecular-Weight Fucoidan as a Supplemental Therapy in Metastatic Colorectal Cancer Patients: A Double-Blind Randomized Controlled Trial †

**DOI:** 10.3390/md15040122

**Published:** 2017-04-21

**Authors:** Hsiang-Lin Tsai, Chi-Jung Tai, Ching-Wen Huang, Fang-Rong Chang, Jaw-Yuan Wang

**Affiliations:** 1 Division of Colorectal Surgery, Department of Surgery, Kaohsiung Medical University Hospital, Kaohsiung Medical University, Kaohsiung 807, Taiwan; chunpin870132@yahoo.com.tw (H.-L.T.); baseball5824@yahoo.com.tw (C.-W.H.); 2 Department of Surgery, Faculty of Medicine, College of Medicine, Kaohsiung Medical University, Kaohsiung 807, Taiwan; 3 Graduate Institute of Natural Product, College of Pharmacy, Kaohsiung Medical University, Kaohsiung 807, Taiwan; taichijung@gmail.com (C.-J.T.); aaronfrc@kmu.edu.tw (F.-R.C.); 4 Department of Family Medicine, Pingtung Hospital, Ministry of Health and Welfare, Pingtung 928, Taiwan; 5 Graduate Institute of Clinical Medicine, College of Medicine, Kaohsiung Medical University, Kaohsiung 807, Taiwan; 6 Center for Biomarkers and Biotech Drugs, College of Medicine, Kaohsiung Medical University, Kaohsiung 807, Taiwan; 7 Research Center for Environmental Medicine, College of Medicine, Kaohsiung Medical University, Kaohsiung 807, Taiwan; 8 Research Center for Natural Products & Drug Development, Kaohsiung Medical University, Kaohsiung 807, Taiwan

**Keywords:** low-molecular-weight fucoidan, metastatic colorectal cancer, supplemental therapy, disease control rate

## Abstract

Background: Low-molecular-weight fucoidan (LMF) is widely used as a food supplement for cancer patients. However, all of the studies are in vitro or were conducted using mice. Therefore, powerful clinical evidence for LMF use is relatively weak. This study aimed to evaluate the efficacy of LMF as a supplemental therapy to chemo-target agents in metastatic colorectal cancer (mCRC) patients. Methods: We conducted a prospective, randomized, double-blind, controlled trial to evaluate the efficacy of LMF as a supplemental therapy to chemotarget agents in patients with metastatic colorectal cancer (mCRC). Sixty eligible patients with mCRC were included. Finally, 54 patients were enrolled, of whom 28 were included in the study group and 26 in the control group. The primary endpoint was the disease control rate (DCR), and secondary endpoints included the overall response rate (ORR), progression-free survival (PFS), overall survival (OS), adverse effects (AEs), and quality of life (QOL). Results: The DCRs were 92.8% and 69.2% in the study and control groups, respectively (*p* = 0.026), in a median follow-up period of 11.5 months. The OS, PFS, ORR, AEs, and QOL did not significantly differ between the two groups. Conclusion: This is the first clinical trial evaluating the efficacy of LMF as a supplemental therapy in the management of patients with mCRC. The results indicate that LMF combined with chemotarget agents significantly improved the DCR.

## 1. Introduction

Fucoidan is an aggregate name for algal fucose-enriched sulfated polysaccharides extracted from the extracellular matrix of brown, green, and red seaweeds [1]. Fucoidan was first introduced by Dr. Kylin in 1913 while analyzing the reason behind the lower incidence of cancer in Okinawa, Japan [2]. Since then, more than 1400 studies focusing on its biological activities have been conducted. In particular, the antioxidant, anticancer, anti-inflammatory, and anti-proliferative activities of fucoidan have attracted considerable attention [3]. Therefore, fucoidan has become a widely used food supplement in Asia, especially in Japan, China, Taiwan, and Australia. The annual production value of fucoidan-related products is more than US $100 million.

Many basic extraction methods of fucoidan products are available, including hot water, acid, ethanol, and alkaline extraction [4]. However, the production rate of fucoidan is low. Therefore, enzyme- [5], microwave- [6], and ultrasound-assisted extraction methods [7] and pressurized liquid extraction were developed [8]. According to different extraction methods, the molecular weight of fucoidan products can range from 20,000 to 200,000 daltons or from 400 to 5000 daltons, which is called low-molecular-weight fucoidan (LMF) [9]. The structure of fucoidan principally consists of an α-1,3-linked or α-1,4-linked backbone, mainly with repeated l-fucose and sulfate units, along with small quantities of d-galactose, d-xylose, d-mannose, and uronic acid [10]. The structure and amount of sulfate groups affect the anticancer effect of fucoidan [11].

Fucoidan and LMF are widely used as a complementary therapy or a food supplement in complementary alternative medicine. Since 2002, more than 200 patients with cancer have been receiving fucoidan as a complementary therapy in the clinic of Dr. Tachikawa Daisuke from Japan [12]. According to case reports in his book, fucoidan had beneficial effects on patients with colorectal, pancreatic, bladder, uterine, lung, liver, breast, and prostate cancers. Most of these patients consumed fucoidan while receiving surgery, chemotherapy, or radiotherapy. These patients received a daily dosage of 1–5 g of fucoidan. Fucoidan reduced the tumor size and the adverse effects (AEs) of chemotherapy and improved the quality of life (QOL). A recent study reported that LMF enhanced the responses of radiotherapy and chemotherapy. In addition, fucoidan reduced AEs and improved QOL. However, these studies were not conducted under strict clinical evaluation and protocols [13].

Two clinical trials have evaluated the efficacy of fucoidan in cancer patients. A randomized trial was performed on 20 advanced or recurrent colorectal cancer patients [14]. Patients were scheduled to undergo FOLFOX6 or FOLFIRI chemotherapy. A high-molecular-weight product of fucoidan, which was derived from *Cladosiphon okamuranus*, was used in the fucoidan group. In the study group, each patient received 150 mL/day of liquid that contained 4.05 g fucoidan for six months from the initial day of chemotherapy. The result showed that fucoidan regulated the occurrence of fatigue during chemotherapy. The second clinical trial demonstrated an open label noncrossover study in breast cancer patients taking letrozole or tamoxifen [15]. Patients took oral fucoidan, derived from *Undaria pinnatifida* extract, for a three-week period (500 mg capsule twice daily). The results suggested that fucoidan in the studied form and dosage could be taken concomitantly with letrozole and tamoxifen, without the risk of clinically significant interactions.

LMF inhibits tumor angiogenesis through the downregulation of HIF-1/VEGF signaling under hypoxia [16], as well as breast cancer cell growth in vitro and in vivo through the involvement of ubiquitin proteasome pathway (UPP)-mediated transforming growth factor-β (TGFβ) receptor degradation [17]. In lung cancer, LMF induced inhibitory activities through the Smurf2-dependent UPP in TGFβ receptor degradation [18]. Likewise, LMF increases the microRNA-29b level to regulate the DNMT3B-MTSS1 axis and inhibits EMT (Epithelial Mesenchymal Transition) in human hepatocellular carcinoma cells [19]. A prospective, open-label, single-arm clinical study was conducted for 20 advanced cancer patients by using LMF (4 g daily in aqueous solution) via oral administration for at least four weeks [20]. The study recruited 20 patients with 10 different origin cancers, including lung, colon, liver, pancreas, stomach, sarcoma, uterus, breast, prostate, head, and neck cancers. The main proinflammatory cytokines, including interleukin-1β (IL-1β), IL-6, and tumor necrosis factor-α (TNF-α) were significantly reduced after two weeks of fucoidan ingestion. 

In Taiwan, colorectal cancer (CRC) has become the most common malignancy in the most recent eight years [21]. In addition, CRC is the third most common cancer in the United States. Personalized treatment protocols have shown beneficial effects in patients. However, the survival rate and QOL of patients with metastatic CRC (mCRC) still require improvement. The development of an efficient auxiliary therapy can be helpful. The previous clinical trial, which applied high-molecular-weight fucoidan in colorectal cancer patients, is a relatively small sample size trial without blindness. Additionally, target therapy should be administrated according to the National Comprehensive Cancer Network (NCCN) guideline. Due to the reason mentioned above, the result of the study couldn’t give us strong evidence for current clinical practice. Therefore, we performed the first prospective, randomized, double-blind, controlled trial to investigate the efficacy of LMF as a supplemental therapy to chemotarget agents in patients with mCRC.

## 2. Materials and Methods

### 2.1. Study Design

This prospective, randomized, double-blind, controlled trial was conducted between December 2014 and August 2016. Initially, we enrolled 60 patients with mCRC and divided them into two groups: study group (*n* = 30) and control group (*n* = 30). In these patients, folinic acid, 5-fluorouracil, and irinotecan (FOLFIRI), plus bevacizumab therapy (5 mg/Kg), was used biweekly as the first-line chemotarget regimen. In the study group, each patient received 4 g of fucoidan BID (*bis in die*, which in Latin means twice a day). In the control group, each patient orally received 4 g of cellulose powder BID. The prescribed period was six months. All clinical data were collected after obtaining informed consent from all patients, and the study protocol was approved by the institutional review board-II of Kaohsiung Medical University Hospital (Identification code: KMUHIRB-2014-09-01[II], approval date: 21 October 2014).

### 2.2. Materials

The low-molecular-weight fucoidan (LMF) powders in this trial were derived from *Sargassum hemiphyllum* and prepared by (Hi-Q Marine Biotech International Ltd. (Taipei, Taiwan), which has a Good Manufacturing Practice certification, and its LMF powder has been qualified as a Symbol of National Quality product in Taiwan. LMF was obtained by enzyme hydrolysis of the original fucoidan. The characteristics of LMF were an average molecular weight of 0.8 KDa (92.1%), fucose content of 210.9 ± 3.3 mmol/g, and sulfate content of 38.9% ± 0.4% (*w*/*w*) [22]. The extraction method followed the method mentioned before, with technological modifications [23]. LMF and cellulose powders were packaged in an aluminum foil bag with the same appearance and weight. Each packet had 4 g of powder in it.

### 2.3. Patient Selection

The inclusion criteria for the study were as follows: (1) age between 20 and 80 years; (2) presence of either metachronous or synchronous mCRC; (3) female patients who were prepared to not breastfeed; (4) no presence of major underlying diseases, such as cardiovascular, cerebrovascular, malignant hypertension, inadequate hematological function, kidney, liver, and other major diseases, or other malignancies; (5) confirmation of mCRC by reports from pathologists or radiologists; and (6) willingness to sign an informed consent form. Patients who did not meet the inclusion criteria or were unwilling to participate were excluded. In addition, patients who could not tolerate regular chemotarget agents or were lost to follow-up within six months were excluded. The demographic and clinical characteristics of the patients were recorded.

### 2.4. Randomization and Blinding

Patients were randomly assigned with an equal probability. A randomization table was created using Microsoft Excel. After inclusion, each patient was assigned a project number, which represented a specific treatment plan. The randomization list was only available to the sample manufacturer; it was unblinded after the completion of the research. 

### 2.5. Study Protocols 

Patients were randomly assigned to study and control groups. Both groups received chemotherapy with targeted therapy according to the National Comprehensive Cancer Network guideline (https://www.nccn.org/). Patients in the study group received 4 g of LMF powder BID for six months. Patients in the control group received 4 g of cellulose powder BID for six months.

All patients underwent laboratory tests, abdominal computed tomography (CT) or other imaging studies, and colonoscopy before the trial. They underwent blood examination and AE evaluation, and filled in a QOL questionnaire every time they were admitted for regular chemotargeted therapy. Adverse events were graded according to the Common Terminology Criteria for Adverse Events (CTCAE), Version 4.02, and the European Organization for Research and Treatment of Cancer (EORTC) QLQ-CR29. The QOL questionnaire was modified from the EORTC QLQ-C30.

### 2.6. Efficacy Objectives

#### 2.6.1. Primary Objective

We selected the disease control rate (DCR) as the primary objective because we focused on the auxiliary effects, and not on the therapeutic effects, of LMF.

#### 2.6.2. Secondary Objectives

We included six secondary endpoints: (1) objective response rate (ORR); (2) overall survival rate (OS); (3) progression-free survival (PFS) rate; (4) incidence; (5) severity of AEs; and (6) QOL.

#### 2.6.3. Safety Objective

If patients experienced adverse events and a laboratory toxicity of more than grade 2 according to the National Cancer Institute CTCAE, the treatment was withheld.

### 2.7. Length of Study

The recruitment period of this study was expected to be 24 months (a median follow-up period of 11.5 months). The trial was continued for six months to reach its primary objective. Survival data were collected until death or a patient’s request for withdrawal.

### 2.8. Efficacy Outcome Measures

The response was assessed radiologically through CT, magnetic resonance imaging, bone scanning, or positron emission tomography, and the most satisfactory response was recorded. The first response was assessed after the sixth cycle in patients who received targeted therapy combined with FOLFIRI chemotherapy. Investigators classified responses according to the Response Evaluation Criteria in Solid Tumors (Version 1.1) [24].

A complete response (CR) is defined as the disappearance of all target lesions. A partial response (PR) is defined as a decrease of at least 30% in the sum of the longest diameter, taking the sum of the longest diameter at the baseline as a reference point. Progressive disease (PD) is defined as an increase of at least 20% in the sum of the longest diameter of target lesions, taking the smallest sum of the longest diameters recorded before the patient started receiving treatment as a reference point. PD can also be defined as the identification of one or more new lesions. Stable disease (SD) is defined as having neither a sufficient shrinkage to qualify for a PR nor a sufficient increase to qualify for a PD. OS is defined as the date of death or the last recorded date of follow-up. Moreover, we reported the best response, which is defined as the best response recorded by the investigators. AEs and AESIs (Adverse Event of Special Interests) were assessed according to the NCI CTCAE, Version 4.03 [25].

### 2.9. Statistical Analysis

All data were analyzed using the Statistical Package for Social Sciences, version 19.0 (SPSS, Inc., Chicago, IL, USA). Data are presented as the mean ± standard deviation. The chi-square test, Wilcoxon rank sum test, and Fisher’s exact test were used to analyze the potential correlation among the expression of biological markers, adverse events, and QOL of patients. OS was defined as the time from the date of primary treatment to the date of death from any cause or until the date of the last follow-up. PFS was defined as the time from the date of primary treatment to the date of diagnosis of PD or to the date of the last follow-up. The cumulative OS and PFS rates were calculated using the Kaplan–Meier method, and differences in the survival rates between the study and control groups were analyzed using the log-rank test. To minimize the interpretation bias, OS analysis was applied. A *p* value of <0.05 was considered statistically significant.

## 3 Results

### 3.1. Baseline Characteristics of Patients

**Figure 1:**
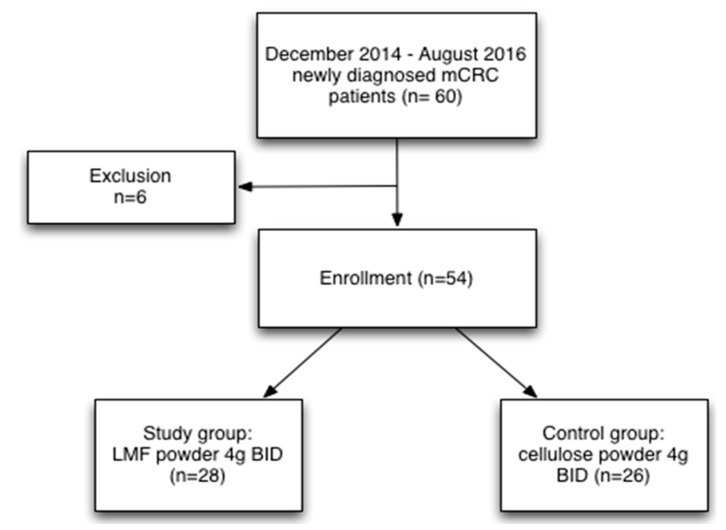
Flowchart illustrating the selection process. Presentation of the cohort and the selection of patients with metastatic colorectal cancer.

From December 2014 to August 2016, sixty patients with a diagnosis of mCRC were included (Figure 1). Of these patients, six could not complete the six-month trial, two had severe AEs, two were lost to follow-up, one died of pneumonia, and one died of severe upper gastrointestinal bleeding. Finally, 54 patients were enrolled, of whom 28 were included in the study group and 26 were part of the control group.

Demographic characteristics, such as sex, age, metachronous mCRC, synchronous mCRC, median follow-up period, metastasectomy, and pretreatment laboratory examinations (white blood cell and platelet counts; hemoglobin, glutamic-pyruvic transaminase, creatinine, and carcinoembryonic antigen levels; and body weight), were similar between the study and control groups (Table 1).

### 3.2. Primary Outcome

The DCR, defined as the sum of the CR, PR, and SD rates, was significantly higher (by 23.6%) in the study group than in the control group (92.8% vs. 69.2%; *p* = 0.026; Table 2).

### 3.3. Secondary Outcomes

**Figure 2:**
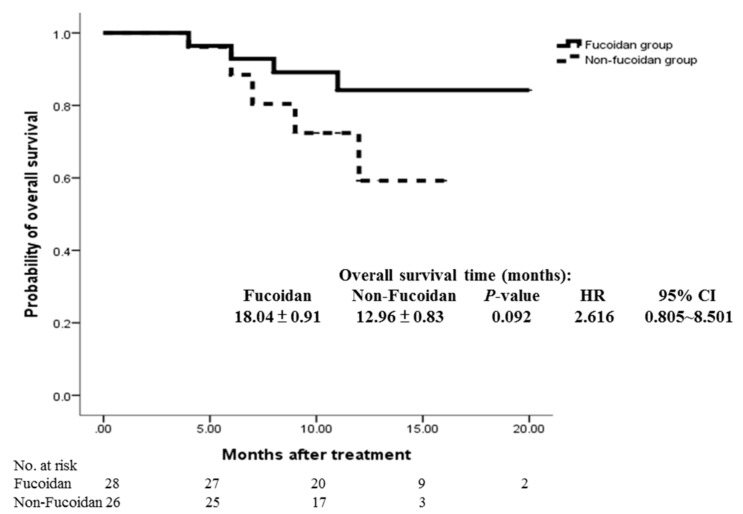
Cumulative overall survival rates of the study and control groups were analyzed using the Kaplan–Meier method, with differences compared using the log-rank test. No significant differences were observed between the two groups (*p* = 0.092).

**Figure 3:**
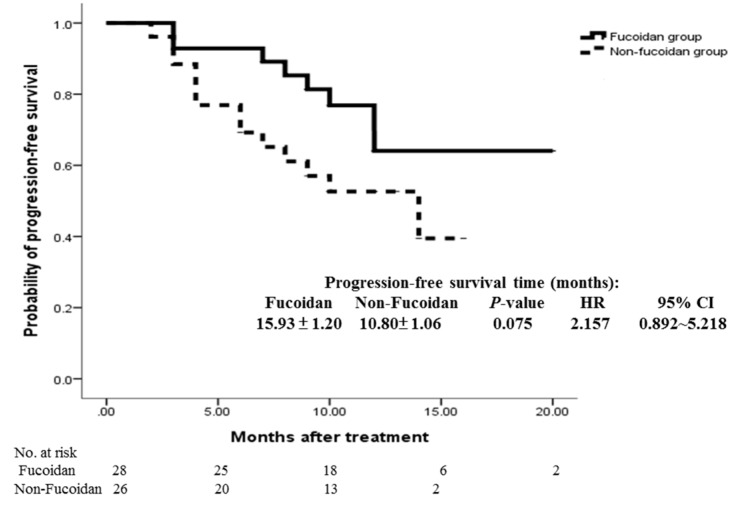
Cumulative progression-free survival rates of the study and control groups were analyzed using the Kaplan–Meier method with differences compared using the log-rank test. No significant differences were observed between the two groups (*p* = 0.075).

The ORR (sum of the CR and PR rates) was comparable in the study group and the control group (60.7% vs. 46.2%; *p* = 0.284; Table 2). Compared with the control group, the study group exhibited a trend of improved OS (18.04 ± 0.91 vs. 12.96 ± 0.83 months; *p* = 0.092) and PFS (15.93 ± 1.20 vs. 10.80 ± 1.06 months; *p* = 0.075); however, the difference was not significant (Figure 2 and Figure 3).

### 3.4. Evaluation of Adverse Effects

No severe AEs were observed in both groups during the trial period. Furthermore, the discontinuation of treatment because of drug-related AEs was not reported. No death was observed to be related to fucoidan treatment. The duration and treatment intensity of the chemotargeted therapy were similar in both groups.

The deflection of all baseline blood examinations levels was similar in both groups (Table 3). The grading of leukopenia, anemia, and thrombocytopenia also exhibited a similar distribution (Table 4). Moreover, the control group exhibited a trend of a higher incidence of oral mucositis (65.4% vs. 50%; *p* = 0.253), pruritus (53.9% vs. 35.7%; *p* = 0.180), vomiting (53.9% vs. 35.7%; *p* = 0.180), taste problem (80.8% vs. 64.3%; *p* = 0.177), and bloody stool (30.8% vs. 14.3%; *p* = 0.145) than the study group; however, the difference was not significant (Table 4).

### 3.5. Quality of Life

Both groups were similar in terms of the limitation of daily activities, limitation of walking, anxiety, fatigue, weakness, and issues of personal hygiene. Grade 3 events including the limitation of pursuing hobbies, trouble sleeping, and depression were not significantly different between the control group and the study group (Table 5).

## 4. Discussion

Approximately 1.2 million patients develop CRC and 600,000 patients die of CRC worldwide annually [26]. In recent years, new anticancer drugs that target oncogenic signaling pathways have been developed; these drugs have demonstrated a prominent efficacy level in the treatment of mCRC [27]. Currently, several EGFR monoclonal antibodies are used for treating mCRC [28]. For example, Erbitux^®^ (cetuximab) and Vectibix^®^ (panitumumab) were approved as first-line treatment for mCRC in 2004 and 2006, respectively [29,30]. Among the major downstream pathways activated by EGFR, the *RAS* mutation played a crucial role in drug resistance [31,32]. *RAS*, *KRAS*, and *NRAS* mutations are detected in approximately 50%, 40%, and 3–5% of patients with CRC, respectively [33,34]. The evaluation of *RAS* mutations has become critical in current clinical practice. Although a personalized treatment plan is effective, the five-year survival rate of patients with mCRC is still approximately 11% [26]. Therefore, developing other therapeutic alternative methods is important to improve the survival rates of patients with mCRC.

Fucoidan and LMF are widely used as a complementary therapy in patients with cancer; nevertheless, no clinical evidence of their efficacy is available. To the best of our knowledge, this is the first clinical trial evaluating the efficacy of fucoidan. In our study, the patients in the study group received 4 g of LMF BID powder for six months. The results reveal that the DCR was significantly higher, with an increase of 23.6% for the study group when compared to the control group. Moreover, the ORR tended to be insignificantly higher in the study group when compared to the control group. A trend of improved OS and PFS was also noted in our analysis. Additional studies with a larger sample size should be conducted to evaluate whether LMF eventually improves OS and PFS. 

According to previous in vitro and animal studies, Fucoidan has a cytotoxic effect in the HCT-15 colon cancer cell line [35]. Fucoidan also inhibits the migration and proliferation of HT-29 human colon cancer cells via the phosphoinositide-3 kinase/Akt/mechanistic target of rapamycin pathways [36]. It was demonstrated that fucoidan represses cancer metastasis by inhibiting vascular endothelial growth factor (VEGF) and matrix metalloproteinases (MMPs) in Lewis tumor-bearing mice [37]. However, the effect of fucoidan in combination with a chemotarget agent has not been evaluated. Furthermore, no scientific report has discussed whether fucoidan can downregulate the EGFR/KRAS/BRAF pathway. It needs further researches to explain the possible anti-cancer mechanism in colon cancer patients.

Previous studies involved clinical observations without systemic evaluations. No study has investigated the hematological effect of fucoidan in vivo. In our study, the grading of leukopenia, anemia, and thrombocytopenia was similar in both groups. Fucoidan has been reported to protect against liver injury and liver fibrosis in mice [38,39]. However, we did not observe this benefit with regard to chemotarget agents. Likewise, LMF could protect renal tubular cells from injury and reduce blood urea nitrogen and creatinine levels in mice [40,41]. In our study, the increase in creatinine levels was similar in both groups; nevertheless, grade III and IV renal impairment levels increased by 11.8% in the control group when compared to the study group, but not significantly. Fucoidan can prevent intestinal mucositis induced by chemotherapy in mice [42]. Our study determined that the study group had a 16.5% lower incidence rate of taste problems than the control group. In an animal model, fucoidan attenuated existing allodynia and hyperalgesia [43].

## 5. Conclusions

This is the first randomized, double-blind, controlled trial evaluating the efficacy of LMF as a supplemental therapy in patients with mCRC. Our study results demonstrate the advantages of LMF in improving the disease control rate (DCR). We believe that this study can provide insights into the development of cancer treatments, particularly in the combination of natural or herbal products with chemotarget agents.

## Figures and Tables

**Table 1:** Clinicopathological features of 54 enrolled patients with stage IV colorectal cancer, comprising 28 patients in the study group and 26 in the control group, evaluated using the chi-square test and Wilcoxon rank sum test.

	Total	Study Group	Control Group	
	(*n* = 54)	(*n* = 28)	(*n* = 26)	*p*-value
	N	N (%)	N (%)	
Gender				0.967 ^#^
Male	31	16 (57.1)	15 (57.7)	
Female	23	12 (42.9)	11 (42.3)	
Age (y/o) *				0.178 ^#^
<65	36	21 (75.0)	15 (57.7)	
≥65	18	7 (25.0)	11 (42.3)	
Age (y/o) *				0.137 ^##^
Median ± S.D *		57.46 ± 12.15	62.38 ± 11.72	
(range)		(30 ~ 79)	(43 ~ 83)	
Stage IV				0.872 ^#^
Synchronous	40	21 (75.0)	19 (73.1)	
Metachronous	14	7 (25.0)	7 (26.9)	
Metastasectomy				0.244 ^#^
Yes	12	8 (28.6)	4 (15.4)	
No	42	20 (71.4)	22 (84.6)	
Follow-up (months)				0.117 ^##^
Median ± S.D *		12.39 ± 4.41	10.54 ± 3.22	
(range)		(4 ~ 20)	(4 ~ 16)	
Pre-WBC ** (/μL)				0.671 ^##^
Mean ± S.D *		7118 ± 2669	7330 ± 2960	
Median		7065	7045	
Pre-Hgb ** (g/dL)				0.472 ^##^
Mean ± S.D *		11.89 ± 1.78	11.51 ± 1.90	
Median		11.80	11.45	
Pre-Platelet (/μL)				0.952 ^##^
Mean ± S.D *		303,964 ± 99,869	300,346 ± 87,945	
Median		278,500	292,500	
Pre-GPT ** (U/L)				0.646 ^##^
Mean ± S.D *		23.54 ± 12.56	20.92 ± 6.80	
Median		21.50	20.50	
Pre-Cr ** (mg/dL)				0.591 ^##^
Mean ± S.D *		0.96 ± 0.75	0.89 ± 0.26	
Median		0.815	0.835	
Pre-CEA ** (ng/mL)				0.236 ^##^
Mean ± S.D *		989.9 ± 3622.5	35.05 ± 60.78	
Median		31.38	13.92	
Pre-Body weight (kg)				0.382 ^##^
Mean ± S.D *		62.39 ± 11.30	59.50 ± 11.90	
Median		61.45	58.00	

^#^ chi-square test; ^##^ Wilcoxon rank sum test; * y/o: years old; S.D.: standard deviation; ** WBC: white blood count; Hgb: hemoglobin; GPT: glutamic-pyruvic transaminase; Cr: creatinine; CEA: carcinoembryonic antigen.

**Table 2:** Comparison of disease control rates and objective response rates between the study and control groups using the chi-square test.

	Total	Study Group	Control Group	
	(*n* = 54)	(*n* = 28)	(*n* = 26)	*p*-value
Disease control rate				0.026
Yes (CR+PR+SD) *	44	26 (92.8)	18 (69.2)	
No (PD) *	10	2 (7.2)	8 (30.8)	
Objective response rate				0.284
Yes (CR+PR) *	29	17 (60.7)	12 (46.2)	
No (SD+PD) *	25	11 (39.3)	14 (53.8)	

* CR: complete response; PR: partial response; SD: stable disease; PD: progressive disease classified using RECIST criteria, Version 1.1.

**Table 3:** Deflection of biochemical indices.

	Study Group (*n* = 28)			Control Group (*n* = 26)			
	Mean	SD	Median	Mean	SD	Median	*p*-value *
WBC change (/μL)	−4508	2444	−4435	−4647.69	2821	−4390	0.8558
Hgb change (g/dL)	−3.00	1.80	−2.95	−2.49	1.77	−2.25	0.3451
Platelet change (/μL)	−144,750	120,530	−92,000	−132,808	86,573	−124,000	0.8152
GPT change (U/L)	75.25	122.61	40.50	58.73	90.57	29.00	0.5974
Creatinine change (mg/dL)	0.52	0.86	0.16	0.69	1.52	0.16	0.2354
CEA change (ng/mL)	−518.90	2472	−12.31	1.41	111.32	−5.56	0.4208
Body weight change (kg)	−3.71	4.27	−2.20	−3.76	4.60	−2.55	0.9035

* Wilcoxon rank sum test; White blood cell (WBC) count change = Minimum WBC count − baseline WBC count; Hemoglobin (Hgb) level change = Minimum Hgb level − baseline Hgb level; Platelet count change = Minimum platelet count − baseline platelet count; glutamic-pyruvic transaminase (GPT) level change = Maximum GPT level − baseline GPT level; creatinine level change = Maximum creatinine level − baseline creatinine level; carcinoembryonic antigen (CEA) level change = last CEA level − baseline CEA level; body weight change = minimum body weight − baseline body weight.

**Table 4:** Comparison of the incidence of adverse effects and severe adverse effects between the study and control groups evaluated using the chi-square test.

	Study Group (*n* = 28) (%)	Control Group (*n* = 26) (%)		Study Group (*n* = 28) (%)	Control Group (*n* = 26) (%)	
Grade	I-IV	I-IV	*p*-value	III & IV	III & IV	*p*-value
Leukopenia	18 (64.3%)	17 (65.4%)	0.9327	7 (25%)	7 (26.9%)	0.8719
Anemia	22 (78.6%)	17 (65.4%)	0.2797	8 (28.6%)	8 (30.8%)	0.8597
Thrombocytopenia	7 (25%)	4 (15.4%)	0.3807	1 (3.6%)	2 (7.7%)	0.5089
Abnormal liver function	14 (50%)	15 (57.7%)	0.5710	5 (17.9%)	4 (15.4%)	0.8075
Impaired renal function	12 (42.9%)	6 (23.1%)	0.1234	1 (3.6%)	4 (15.4%)	0.1346
Mucositis oral	14 (50%)	17 (65.4%)	0.2533	1 (3.6%)	1 (3.8%)	0.9574
Pruritus	10 (35.7%)	14 (53.9%)	0.1803	0	0	
Vomiting	10 (35.7%)	14 (53.9%)	0.1803	0	3 (11.5%)	0.0644
Taste problem	18 (64.3%)	21 (80.8%)	0.1766	2 (7.1%)	2 (7.7%)	0.9386
Bloody stool	4 (14.29%)	8 (30.77%)	0.1454	0	1 (3.85%)	0.2948
Alopecia	26 (92.9%)	25 (96.1%)	0.5971	10 (35.7%)	9 (34.6%)	0.9326

Severe adverse effects: grade 3 and 4 adverse effects. Grading modified from the Common Terminology Criteria for Adverse Events, Version 4.02, and the European Organization for Research and Treatment of Cancer QLQ-CR29.

**Table 5:** Quality of life grading between the study and control groups during treatment by using Fisher’s exact test.

	Study Group (*n* = 28)				Control Group (*n* = 26)				
Grading	0	1	2	3	0	1	2	3	*p*-value
Limited in doing daily activities	0	12	13	3	0	10	10	6	0.5142
Limited in doing hobbies	4	15	6	3	0	11	13	2	0.0553
Limited in walking	9	14	3	2	6	9	9	2	0.2197
Trouble sleeping	7	12	6	3	4	5	14	3	0.0784
Depression	7	15	6	0	1	16	8	1	0.0971
Anxiety	6	12	9	1	3	12	11	0	0.5826
Fatigue	3	12	11	2	0	11	12	3	0.4520
Feel weakness	1	15	9	3	0	9	14	3	0.3108
Need help with personal hygiene	18	5	4	1	12	10	2	2	0.3031

Quality of Life evaluated using the European Organization for Research and Treatment of Cancer (EORTC) QLQ-CR30.

## Supplementary Materials

The following are available online at www.mdpi.com/1660-3397/15/4/122/s1, Table S1: The definitions of adverse effects grading, Table S2: The definitions of quality of life.
