# Supplementary material for: Efficacy of Low-Molecular-Weight Fucoidan as a Supplemental Therapy in Metastatic Colorectal Cancer Patients: A Double-Blind Randomized Controlled Trial
Source: Mar Drugs. 2017 Apr 21;15(4):122. doi: 10.3390/md15040122 (PMC5408268; doi:10.3390/md15040122)
Supplement: Supplementary file 1 [file marinedrugs-15-00122-s001.pdf]

Efficacy of Low-Molecular-Weight Fucoidan as a Supplemental Therapy in Metastatic Colorectal Cancer Patients: A Double-Blind Randomized Controlled Trial

Hsiang-Lin Tsai [1,2,†], Chi-Jung Tai [3,4,†], Ching-Wen Huang [1,2], Fang-Rong Chang [3] and Jaw-Yuan Wang [1,2,5,6,7,8,*]

[1] Division of Colorectal Surgery, Department of Surgery, Kaohsiung Medical University Hospital, Kaohsiung Medical University, Kaohsiung 80708, Taiwan; chunpin870132@yahoo.com.tw(H.L.T); baseball5824@yahoo.com.tw (C.W.H);
[2] Department of Surgery, Faculty of Medicine, College of Medicine, Kaohsiung Medical University, Kaohsiung 80708, Taiwan
[3] Graduate Institute of Natural Product, College of Pharmacy, Kaohsiung Medical University, Kaohsiung 80708, Taiwan; taichijung@gmail.com (C.J.T); aaronfrc@kmu.edu.tw (F.R.C)
[4] Department of Family Medicine, Pingtung Hospital, Ministry of Health and Welfare, 80708, Taiwan
[5] Graduate Institute of Clinical Medicine, College of Medicine, Kaohsiung Medical University, Kaohsiung 80708, Taiwan
[6] Center for Biomarkers and Biotech Drugs, College of Medicine, Kaohsiung Medical University, Kaohsiung 80708, Taiwan
[7] Research Center for Environmental Medicine, College of Medicine, Kaohsiung Medical University, Kaohsiung 80708, Taiwan
[8] Research Center for Natural Products & Drug Development, Kaohsiung Medical University, Kaohsiung 80708, Taiwan
* Correspondence: jawyuanwang@gmail.com (J.Y.W); Tel.: +886-7-3122-805, Fax: +886-7-3114-679
† The authors have contributed equally to this work.

Supplementary table 1. The definitions of adverse effects grading according to Common Terminology Criteria for Adverse Events (CTCAE) version 4.02

Grading	0	1	2	3	4
Leukopenia	>3000	2501-3000	2001-2500	1001-2000	<1000
Anemia	>10	9-10	8-9	6.5-8	<6.5
Thrombocytopenia	>100000	75000-100000	50000-75000	25000-50000	<25000
Abnormal liver function	Below upper limit	1-2 X upper limit	2-3 X upper limit	3-5 X upper limit	>5 X upper limit
Impaired renal function	increase < 0.3 mg/dl over baseline	1.5 -2X baseline or increase >0.3 mg/dl over baseline	2-3 X baseline	>3 X baseline or Cr > 4 mg/dL	Need immediate dialysis
Mucositis oral	No lesion	Asymptomatic or mild symptoms; intervention not indicated	Moderate pain; not interfering with oral intake; modified diet indicated	Severe pain; interfering with oral intake	Life-threatening consequences; urgent intervention indicated
Vomiting	No symptoms	Mild symptoms	Moderate symptoms; frequent	Persisted; very much	-
Alopecia	No hair loss	Not obvious from close inspection	Hair loss of up to 50% . That is not obvious from a	Hair loss >50%	Total hair loss

			distance but only on close inspection		
Pruritis		Mild or localized; topical intervention indicated	Intense or widespread; Intermittent; Skin change, need oral medication.	Intense or widespread; constant; oral corticosteroid indicated	-
Bloody stool	No symptom	Mild symptom	Moderate symptom	Severe symptom; anal bleeding	Urgent intervention indicated
Taste problem	No symptom	A little	Quite a bit	Very much	urgent intervention indicated

Supplementary table 2. The definitions of quality of life grading according to European Organization for Research and Treatment of Cancer (EORTC) QLQ-CR29 and QLQ-CR30

Grading	0	1	2	3
Limited in doing daily activities	No limit	A little	Quite a bit	Very much
Limited in doing hobbies	No limit	A little	Quite a bit	Very much
Limited in walking	No limit	A little; in long distance	Quite a bit; in short distance	Very much
Trouble sleeping	No symptom	Mild symptom	Moderate symptom; Need hypnotics	Severe symptom; No improvement under hypnotics
Depression	No symptom	Mild depressive symptoms	Moderate depressive symptoms; limiting instrumental ADL	Severe depressive symptoms; limiting self care ADL;
Anxiety	No symptom	Mild symptoms; intervention not indicated	Moderate symptoms; limiting instrumental ADL	Severe symptoms; limiting self care ADL; hospitalization notindicated
Fatigue	No symptom	A little	Quite a bit	Very much

Feel weakness	No symptom	A little	Quite a bit	Very much
Need help with personal hygiene	No limit	A little	Quite a bit	Very much
